# Analysis of Cushioned Landing Strategies of Cats Based on Posture Estimation

**DOI:** 10.3390/biomimetics9110691

**Published:** 2024-11-13

**Authors:** Li Zhang, Liangliang Han, Haohang Liu, Rui Shi, Meiyang Zhang, Weijun Wang, Xuyan Hou

**Affiliations:** 1 Research Center of Aerospace Mechanism and Control, School of Mechatronics Engineering, Harbin Institute of Technology, Harbin 150080, China; hollyoneone@163.com (L.Z.); liuhaohang5000@163.com (H.L.); 13339465939@163.com (R.S.); zhangmeiyanghit@163.com (M.Z.); 2 Space Structure Mechanism Technology Laboratory, China Aerospace Science and Technology Group Co., Ltd., Shanghai 201109, China; 3 Zhengzhou Research Institute, Harbin Institute of Technology, Zhengzhou 450000, China; 4 School of Mechanical Engineering and Automation, Beihang University, Beijing 100083, China; hllrob@163.com; 5 Aerospace System Engineering Shanghai, Shanghai 201109, China; 6 Shanghai Institute of Aerospace System Engineering, Shanghai 201109, China; 7 Songjiang Laboratory, Harbin Institute of Technology, Harbin 150080, China

**Keywords:** crawling space robot, minimizing landing impact, animal posture estimation, cat’s landing strategy

## Abstract

This article addresses the challenge of minimizing landing impacts for legged space robots during on-orbit operations. Inspired by the agility of cats, we investigate the role of forelimbs in the landing process. By identifying the kinematic chain of the cat skeleton and tracking it using animal posture estimation, we derive the cushioning strategy that cats use to handle landing impacts. The results indicate that the strategy effectively transforms high-intensity impacts into prolonged low-intensity impacts, thereby safeguarding the brain and internal organs. We adapt this cushioning strategy for robotic platforms through reasonable assumptions and simplifications. Simulations are conducted in both gravitational and zero gravity environments, demonstrating that the optimized strategy not only reduces ground impact and prolongs the cushioning duration but also effectively suppresses the robot’s rebound. In zero gravity, the strategy enhances stable attachment to target surfaces. This research introduces a novel biomimetic control strategy for landing control in the on-orbit operations of space robots.

## 1. Introduction

With the development of space technology, higher requirements have been proposed for space in-orbit services [1,2,3]. Crawling space robots, serving as crucial agents for in-orbit services, can provide in-orbit service activities for many satellites, space stations, and other facilities [4,5,6,7]. These robots face challenges with various terrain surfaces as they are unstructured, with high complexity. There is low recognition in space, and the robots need to move agilely, like animals. Despite advancements, current robots cannot perform the same as animals in the real world [8,9,10]. Animals have evolved organs and habits to fit the unpredictable natural environment. The kind of structure and movement strategy evolved by organisms offers great potential for the technological advancement of space robotics [11,12,13,14]. The development of and research into crawling space robot technology have garnered significant global attention. For instance, NASA JPL developed the Hedgehog robot, with a three-axis reaction wheel system to spin-up and somersault, which can creep over steep and uneven obstacles [15,16]. ETH Zürich proposed a cat-like robot that can jump agilely to investigate the potential of dynamic jumping to get around in low gravity environments [17,18]. The Robotic Innovation Center in Bremen presented a walking robot called SCORPION, which uses an analysis of the motion patterns of real scorpions to optimize the biomimetic control scheme [19]. Researchers at Yale University built a robot inspired by terrestrial and aquatic turtles, that fuses traditional rigid components and soft materials to radically augment the shape of its limbs and shift its gait for multi-environment locomotion [20]. Beijing Institute of Technology developed a small-sized quadruped robotic rat, which includes four limbs and one flexible spine, achieving superior motion performance [21]. Beihang University describe an aerial–aquatic hitchhiking robot that is self-contained for flying, swimming, and attaching to surfaces in both air and water, and that can seamlessly move between the two [22]. Harbin Institute of Technology propose a robot capable of moving on trusses and manipulating parts, inspired by the Dynastes Hercules beetle [23,24].

While providing on-orbit services, the robot needs to land precisely on the target location of the space facility to execute its tasks. Excessive landing impact may damage the target surface and the internal circuitry of the robot, thereby affecting the service lifetime of both the space facilities and the robot. The robot needs to minimize the landing impact. However, a space robot is a complex multi-body system. Its motion interacts with the space facilities, influenced by the dynamics of attached cushioning. Collision occurs between the robot’s end effector and the target surface during the landing process. The velocity changes suddenly from a nonzero value to zero in a short time, altering the equation of state of the entire system. There are discontinuous and nonlinear changes in this process. It is a complex system with multiple inputs and multiple outputs, which is highly nonlinear and strongly coupled. Meanwhile, the space robot must contend with the challenges of the space environment, including zero gravity. It is crucial for the robot to successfully land on the target surface without damaging it or disturbing the facility’s orbit. Many researchers have been engaged to tackle these issues. Nikita et al. trained a neural network to control a jumping quadruped robot while solely using its limbs for attitude control. The results demonstrate that the robot can easily generalize across space tasks and land with natural agility [25]. Ji Qi et al. discussed the integrated attitude and landing control scheme for quadruped robots in an asteroid landing mission scenario. By deploying trained reinforcement learning algorithms, the quadruped robot can automatically reposition itself to the most suitable landing attitude based on gravity and terrain information [26]. Yoshida et al. proposed an impedance-matching control method for space robots in the acquisition process of non-cooperative targets. By selecting the posture, the virtual mass can be altered, and the impedance control is introduced to realize a wide range of impedance characteristics [27]. Chi Zhang et al. developed a frog robot with torsional springs as an energy storage device. With the addition of an extending feasible robotic joint trajectory using the La Salle invariant set principle, a trajectory controller was obtained for realizing smooth and stable trajectory tracking in joint space [28].

As mentioned above, the robots should have the ability to traverse challenging terrain, and animals in nature have the potential to be bionic objects. Bingcheng Wang et al. developed a hybrid pneumatic–electric-driven climbing robot with active attachment–detachment bionic flexible feet inspired by the active attachment–detachment behavior of geckos [29]. Qiyuan Cao et al. introduced RobDact, a biomimetic underwater vehicle inspired by the Dactylopteridae, and developed techniques for hydrodynamic modeling and parameter identification [30]. Feng Ding et al. designed the bionic leopard cabinet underwater robot to investigate trajectory planning and tracking control under multiple dynamic obstacles [31]. By imitating movement strategies from animals, like walking, leaping, or crawling, robots can gain new functionality [32,33]. It has been noticed that cats and many other animals can control their body and legs gracefully, which allows them to land safely back on their feet. Zhiqiang Zhang et al. describe the typical landing sequences in which the cat fully extends its back at the beginning of a touchdown. After the forepaws make contact, elbow flexion and shoulder extension occur, while the back remains extended. Then, the cat bends its back as the hindlimbs flex toward its body [34]. It demonstrates that the cat can actively modulate its joint and back muscle stiffness to absorb the optimal kinetic energy of each landing, covering a range of heights. The robots may also learn to extend their legs to do complex tasks like safely landing on the space station. However, tracking cats’ posture is not a simple task. Motion capture systems (e.g., Xsens, Innalabs, and Vicon) based on commercial markers provide high-precision posture recognition [35,36]. There are difficulties in using these commercial systems in mammals. Their dense, colorful fur blends into the environment, creating a great deal of shelter and reducing visibility [37]. Their highly flexible skin makes markers shift position relative to the bone structure during movement. One good solution is to use machine learning algorithms. Recently, the application of machine learning algorithms, such as neural networks, has greatly advanced the state of image-based data processing tasks across the fields of animal behavior and neuroscience. Many deep learning-based tools have been built for animal pose estimation and tracking, which make it possible to observe and analyze cat posture. For example, Talmo D. Pereira et al. presented SLEAP to estimate and track animal poses across flies, bees, mice, and gerbils. The system enables versatile workflows for data labeling, model training, and inference on previously unseen data [38]. Gabriella E. C. Gall et al. found SLEAP to be accurate in tracking the plant behavior of Arabidopsis, Bean, and Sunflower, requiring only a low number of user-labeled frames for training [39]. Jessy Lauer et al. built an open-source pose estimation toolbox called DeepLabCut and provide high-performance animal assembly and tracking—features required for multi-animal scenarios. The power of the framework is demonstrated in mouse culture [40]. Using DeepLabCut, Shota Hayakawa et al. successfully labeled the six body parts of a single cricket with a high level of confidence and produced reliable data showing the diurnal rhythms of multiple behaviors [41]. Jacob M Graving et al. introduced DeepPoseKit, a toolkit that can solve the limitations of animal pose estimation in speed and robustness. The toolkit, verified on locust tracking, showed that it can achieve fast inference without reducing accuracy or generalization ability [42]. Praneet C. Bala et al. describe a motion capture system called OpenMonkeyStudio for estimating 3D poses in free movement. The result showed that the system can be used to accurately recognize actions and track social interactions [43].

This paper proposes a highly adaptive cushioning strategy for space robots to minimize landing impact during on-orbit operations. Inspired by the cat’s landing process, the skeletal structure and motion characteristics are analyzed. With the animal posture estimation framework SLEAP, the trajectory coordinates of various nodes of the forelimbs are obtained. By analyzing the cushioning actions and variations in joint angles, we adapt the cushioning strategy for the robotic platform through reasonable assumptions and simplifications. Simulations are performed in both gravitational and zero gravity environments, indicating that the strategy can not only reduce the ground impact and prolong the cushioning duration but also effectively suppress the robot’s rebound. Moreover, the strategy exhibits high adaptability to the different motion parameters of the robot. The proposed cushioning strategy holds significant implications for the development and enhancement of the on-orbit operations of future space robots.

## 2. Materials and Methods

### 2.1. Cat Posture Estimation

#### 2.1.1. Kinematic Chain of Cat Skeleton

The cats in nature, having evolved over millions of years, can perform agilely due to their unique structural characteristics. They have evolved to move gracefully on any kind of terrain, using four limbs in coordination with their body movement. Locomotion involves repetitive movements and is often executed unconsciously and automatically [44]. The details of this movement can provide a rich stream of information about the interaction between the animal and its surroundings. However, only a fraction of the available information about body movement is utilized in the robot design. For example, for patrolling, jumping, and running, each measure a very limited range of motion from a single modality.

**Figure 1:**
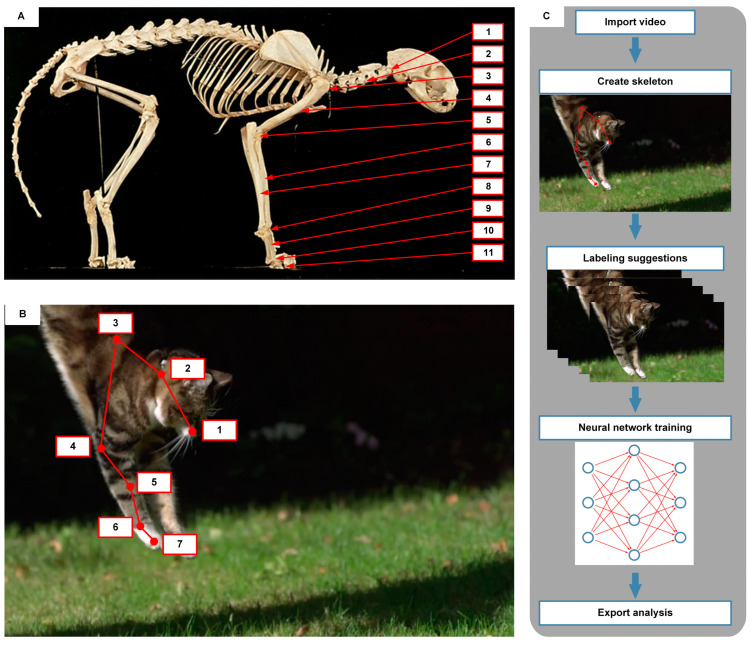
Design and workflow of cat posture estimation. (**A**) Skeleton of cat (right lateral view): 1—neck, 2—cervical vertebrae, 3—shoulder joint, 4—humerus, 5—elbow joint, 6—radius, 7—ulna, 8—wrist joint, 9—metacarpal bones, 10—digital joint, 11—phalanges; (**B**) Kinematic chain of cat skeleton: 1—nose node, 2—neck node, 3—shoulder node, 4—elbow node, 5—wrist node, 6—digital node, 7—toe node; (**C**) Labeling process of SLEAP.

This article focuses on the posture changes of the cat’s forelimbs during the landing process to establish the cushioning strategies of the robot. The skeleton of the cat is depicted in Figure 1A. Cats are observed to be one of the representative animals exhibiting both flexibility and strength. The bones from the head to the end are cervical vertebrae, humerus, radius, ulna, metacarpal bones, and phalanges, while the joints are composed of the shoulder joint, elbow joint, wrist joint, and digital joint. Moreover, the forearm is composed of the radius and ulna, which connect the elbow joint and wrist joint to achieve both pitching and twisting motions. The rest of the forelimbs consist of single bones. Cats have digitigrade feet, walking on their phalanges without the heel touching the ground. The fronts of the phalanges have evolved into sharp and strong toenails, enhancing cats’ hunting efficiency.

As the cat jumps to the ground, the forelimbs exhibit little embracing or grasping movements. Therefore, the ulna and metacarpal bones are treated as a single unit, referred to as the forearm in this article. Seven nodes are selected, starting from the nose and passing through the neck, shoulder, elbow, wrist, digital, and toe ends. Six edges are used to connect the nodes, representing the head, cervical spine, humerus, forearm, metacarpal, and phalanges. These nodes and edges form the kinematic chain of the cat’s forelimb, as illustrated in Figure 1B.

#### 2.1.2. Toolkit of Posture Estimate

SLEAP is an open-access frame for animal posture estimation. It can be used to track any type or number of animals. SLEAP has modular neural network architectures that enable a fast and flexible paradigm. Studies have shown that SLEAP can estimate faster without sacrificing accuracy. Developed entirely in Python 3, SLEAP has a purpose-built GUI and human-in-the-loop workflow for rapidly labeling large datasets. GPU acceleration is supported for efficient and rapid animal posture tracking.

Tracking the cat’s posture as it leaps to the ground, the collision occurs between its foot and the ground. Upon impact, the cat’s skin and fur drift. The cat’s colorful and concealed fur visually blends with the surroundings, and the enrichment objects may occlude the cat from the camera. These factors greatly increase the difficulty of cat pose marking. SLEAP can leverage variability in body morphology and fur patterning across animals as distinguishing features, and estimate each video frame using a Convolutional Neural Network (CNN). There are no temporal dependencies across frames, thereby guaranteeing that nodal drift does not propagate over time. This toolkit can improve the accuracy of posture estimation and reduce the prediction time. Therefore, SLEAP has been selected as the toolkit for posture estimate.

#### 2.1.3. Labeling Process

A 17-second video clip online with a 960 frames per second (fps) shooting frame rate was selected. The clip records the cat jumping from the tree to the ground with a high-speed lens [45]. The edge of the video frame has distortion under the influence of perspective and light. Distortion in videos typically occurs at the edges, ranging from 1% to 5%. The video frames have been preprocessed by Capcut, with 10% cropped from the edges to ensure correction. The preprocessed clip is a 2880*2160-pixel video with 508 frames and 30 fps.

Figure 1C shows the SLEAP workflow to estimate the landing process. After the video is imported, the nodes and edges are set according to the kinematic chain of the cat’s skeleton. Then, 100 evenly spaced samples are taken as candidate groups of images from the video, and 25 evenly spaced frames are taken to be labeled from these samples. As the cat is in the landing process, the body posture changes dramatically. This period has a higher density of information stream than other time periods. Therefore, 25 frames were selected to be labeled, additionally. The cat’s movements in the 50 selected frames (25 evenly spaced frames and the additional 25 frames) were labeled manually. These frames are regarded as training datasets, and the remaining 50 frames are used as test sets. SLEAP trains the model on the datasets and makes predictions on the test sets. After the initial prediction of all 100 frame samples, the labeling results for each frame in the sample are checked and adjusted. This process is repeated to enter the human-in-the-loop training cycle, in which SLEAP continuously receives new video frames and continuously predicts the results of each frame until the dataset reaches the desired posture estimate labeling accuracy. Finally, the labeled dataset is exported as an h5py package, which can be imported into Python 3 for subsequent data analysis.

The detailed labeling settings are in Supplementary Material Section S1. The labeled video is shown in Viedo S1. The entire process is trained using an NVIDIA Tesla P40 24 GB graphics processing unit (GPU). The total training time is about 3 h 20 min.

#### 2.1.4. Data Process

The labeled data should be further processed using Python 3 to analyze the posture changes of the cat’s forelimbs during the landing process. The position data of all marked nodes from the h5py package are exported. The Savitzky–Golay filter is then used to smooth and differentiate data to obtain the pixel locations (px) and velocities (pixel per frame, px/f) of each node in the video clip. The Savitzky–Golay filter is a low-pass filter for smoothing noisy data. It uses polynomial fitting through a moving window to achieve optimal fitting via the least squares method, and it can differentiate data without altering the signal’s trends or widths. In this paper, the moving window size for the filter is set to 9, and the polynomial fit is of the third order.

Additionally, it is crucial to obtain the joint angle variables during the landing process. Six joint angles are calculated using the nodes’ location data, as shown in Supplementary Material Section S2. Similarly, the Savitzky–Golay filter is applied to the joint angle data, extracting both the joint angles (°) and joint angular velocities (°/f).

### 2.2. Robot Simulation

#### 2.2.1. Robotic Simulation Platform

**Figure 2:**
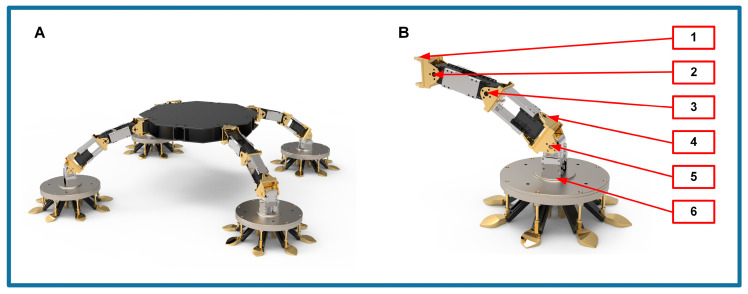
The robotic simulation platform. (**A**) Schematic diagram of the robot; (**B**) Structure of the robot leg: 1—shoulder yaw joint, 2—shoulder pitch joints, 3—elbow joint, 4—wrist pitch joints, 5—wrist yaw joints, 6—SCRBP.

The robotic simulation platform, as shown in Figure 2A, is mainly designed to investigate the cushioning characteristics of the robot in the space environment. The robot features a quadrupedal circular configuration, and the arrangement of its leg joints is inspired by cats, including shoulder yaw joints, shoulder pitch joints, elbow joints, wrist pitch joints, and wrist yaw joints. Each joint is equipped with an actuator to support the robot’s movement requirements, as shown in Figure 2B. The end of each robot leg is equipped with passive buffering feet called SCRBP, which can detect contact and buffer for a reliable connection between the robot and the target surface in space [46]. 

The landing process mainly happens on the sagittal plane of the cat. For the leg joints of cats, the shoulder yaw joint is principally used for the forward movements, while the wrist yaw joint is mainly employed during hunting or feeding to grasp objects. These movements occur in the horizontal and coronal planes, respectively. Therefore, the shoulder pitch joint (short for shoulder joint), elbow joint, and wrist pitch joint (short for wrist joint) are selected as primary subjects of study to examine the robot’s movements within the sagittal plane. The robot needs to sense and utilize the drive of the leg joints to learn the buffering strategies employed by cats when jumping to the ground.

#### 2.2.2. Simulator Selection

MuJoCo, a physics engine optimized for fast and accurate multi-joint robot dynamics, features a convex soft contact model and a scalable API, which is ideal for robotics, biomechanics, graphics, machine learning, and other applications requiring precise simulations [47]. MuJoCo computes forward and inverse dynamics in continuous time. The end result of forward dynamics is the joint accelerations and the actuator forces. These are used to advance the simulation time and update the state variables. There are four types of built-in numerical integrators available for user applications. Among them, the RK4 integrator, which is suitable for its high simulation accuracy and good stability, was chosen. It demonstrates excellent convergence and computational efficiency, especially when dealing with scenarios in robotic multibody systems. Regarding the actuators, it provides a flexible actuator model, with three components that can be specified independently. Together they determine how the actuator works. The preset position and velocity controller are used to simulate the actuator’s behavior.

#### 2.2.3. Simulation Process

The simulation process of MuJoCo comprises three key steps: importing the robot model, loading the simulation environment, and controlling the model movement. First, the various components of the robot platform need to be disassembled and converted into usable robot model parts within their respective sub-coordinate systems (the STL format is used in this paper). Secondly, an XML file is created to reassemble these robot model components. Once the robot is reconstructed in the XML file, its physical properties, such as material, density, stiffness, and damping, are defined. Additionally, the corresponding scenario profile is added. Finally, a Python 3 script is written to load the XML simulation environment and manage the simulation steps. This script continuously advances the simulation through a loop, allowing for control actions as needed, including setting joint target locations, adding external forces, etc. Then the simulation data is exported as an XLSX file. 

In the simulation environment we constructed, the robot is initially positioned above the surface. The robot is set to free fall toward the target surface from a predetermined height to achieve the desired impact velocity. A PD controller is set at the joint of the robot (Supplementary Material Section S3). Contact force sensors are installed at the robot’s foot ends, and velocity sensors are placed on the robot’s main body to measure the impact forces experienced by these parts. The detailed simulation settings are in Supplementary Material Section S4. By utilizing the specific joint control logic, the robot mimics the buffering action of a cat jumping down from a tree until its overall speed is reduced to zero. 

## 3. Results

### 3.1. Analysis of Kinematic Characteristics

**Figure 3:**
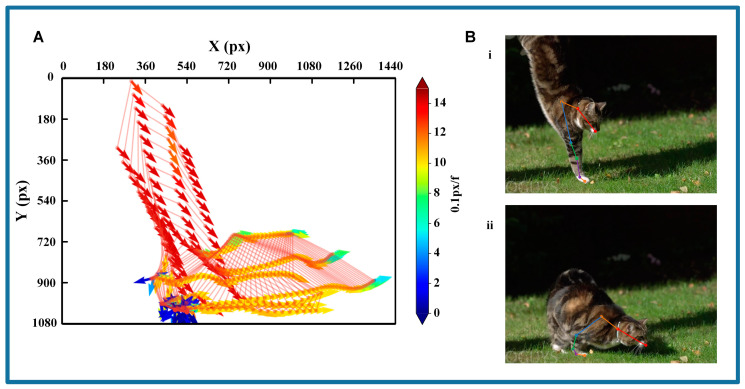
The result of the cat’s landing process. (**A**) The variations in position and velocity in the video clip using SLEAP; (**B**) Two critical frames during contact with the ground: i—the frame as the cat makes contact with the ground; ii—the frame as the cat begins to recover its posture after the landing process.

The cat leaps to the ground under gravity, whereas the space robot operates in a micro-gravity or zero gravity environment. To effectively transfer the cat’s cushioning strategies to the robot platform, a detailed decomposition of the cat’s landing process is required. Figure 3A illustrates the variations in position and velocity in the video clip using SLEAP. The position of each point shows the location of the node, while the color and orientation denote the velocity vector of the joints at that frame. 

It is observed that, during the landing process, the cat transitions from descending toward the ground to moving forward. Drastic changes in velocity and direction occur upon contact with the ground, which also alters the body posture. Thus, two critical frames are identified in Figure 3B. The first frame is defined as the point at which the cat reaches the lowest position and begins to make contact with the ground. The second frame is defined as the moment when the joints start to reverse, marking the conclusion of the landing and the resumption of a walking posture. Consequently, the landing process is divided into three sequential phases. The first phase is the airborne phase, during which the cat leaps from a tree and falls under gravity. While airborne, the cat prepares its body for landing until contact, initiating its buffering strategy (frames 1–97). The second phase is the cushioning phase, characterized by the cat squatting under the impact of landing until its body is fully crouched and the distal joints begin to reverse (frames 98–211). The third phase is the recovery phase, during which the cat stands up and resumes its normal walking posture until the end of the video (frames 212–508). 

During the labeling process, a video of 508 frames was selected to enhance sample size and accuracy in SLEAP. The airborne phase and cushioning phase were selected to investigate the buffering strategy. The proportions of the two phases are 45.97% and 54.03%, respectively. By the recovery phase, the cat has completed the entire buffering process and commenced walking in a regular alternating-step pattern.

#### 3.1.1. Airborne Phase

**Figure 4:**
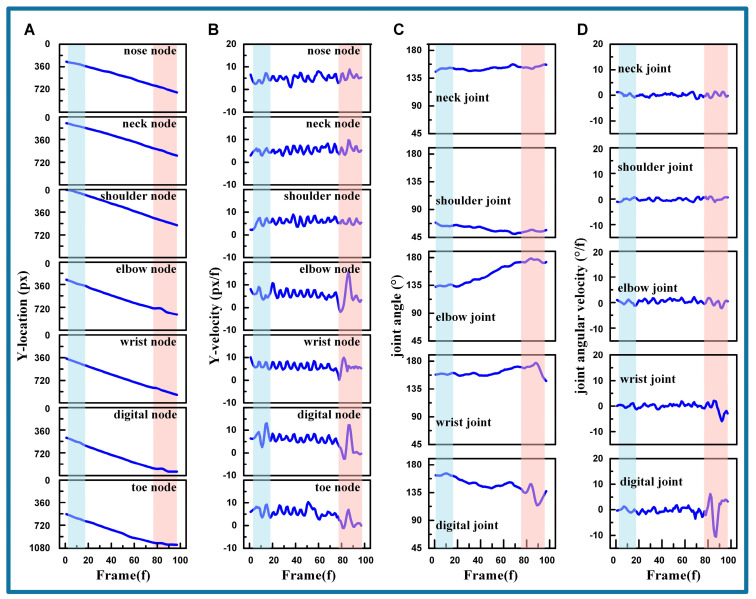
The cat’s kinematic characteristics during the airborne phase. (**A**) The vertical positions of each node. (**B**) The vertical velocities of each node. (**C**) The joint variation of each joint. (**D**) The angular velocities of each joint. (The blue blocks represent frames 1–18, while the red blocks represent frames 78–97).

The analysis of the cat’s kinematic characteristics during the airborne phase (frames 1–97) is illustrated in Figure 4. Specifically, Figure 4A presents the vertical positions of each node as the cat descends. The ordinate values of these nodes’ positions generally increase with each frame. Upon the termination of the airborne phase, and just before the cat reaches the ground (frames 78–97), reverse fluctuations occur in the toe node, the digital node, the wrist node, and the elbow node, while the positions of the other three nodes remain stable. Figure 4B captures the velocities of these nodes. When the cat leaps from the tree (frames 1–18), the velocities of the toe node, the digital node, and the elbow node vary under the influence of inertia. Following this, velocities remain stable until they undergo significant changes as the cat approaches the ground (frames 78–97), corresponding to the patterns observed in Figure 4A. 

As depicted in Figure 4C, it can be observed that the neck joint and shoulder joint have relatively small changes, with their ranges being $151.14_{-5.65}^{+6.21}$° and $58.55_{-7.92}^{+10.33}$°, respectively. In contrast, the elbow joint, wrist joint, and digital joints experience larger variations during descent, with ranges of $154.49_{-22.14}^{+23.79}$°, $162.45_{-14.59}^{+14.68}$°, and $147.28_{-33.14}^{+18.52}$°. With respect to the wrist joint and digital joint just before ground contact (frames 78–97), they exhibit oscillations in the opposite direction to their positional changes, indicating the joints’ preparation for landing. Figure 4D records the angular velocities of each joint, which remain stable over most frames within a certain range, represented by $0.06_{-1.74}^{+1.99}$°/f, $-0.02_{-1.64}^{+3.41}$°/f, $-0.02_{-5.67}^{+5.00}$°/f, $-0.11_{-7.17}^{+5.24}$°, and $-0.18_{-10.49}^{+14.32}$°/f. It can be seen that the amplitude of angular velocity increases gradually from the neck joint to the digital joint. Additionally, when the cat’s paw falls near the ground (frames 78–97), the angular velocities of the wrist and digital joints undergo significant changes. 

According to the analysis above, combined with the video clip of the cat, the cat’s movement is initially maintained as it leaps from the tree, after which it gradually extends its joints in the air to increase the cushioning distance in preparation for landing. Just before the cat’s paw touches the ground, the digital joint and the wrist joint are adjusted to modify the foot posture to align with the horizontal ground. Remarkably, compared to other joints, the digital joint and wrist joint are more active and constantly adjust to finely tune the cat’s front leg posture in the airborne phase. 

#### 3.1.2. Cushioning Phase

**Figure 5:**
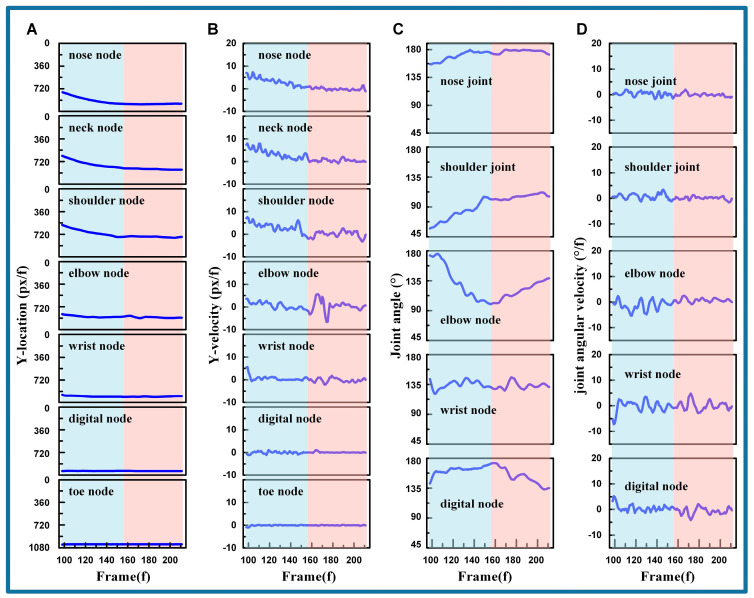
The cat’s kinematic characteristics during the cushioning phase. (**A**) The vertical positions of each node. (**B**) The vertical velocities of each node. (**C**) The joint variation of each joint. (**D**) The angular velocities of each joint. (The blue blocks represent frames 98–156, while the red blocks represent frames 157–211).

The analysis of the cat’s kinematic characteristics during the cushioning phase (frames 98–211) is illustrated in Figure 5. The vertical locations of each node are depicted in Figure 5A. The ordinate values of these nodes’ positions gradually increase with the progression of video frames. Meanwhile, the nodes remain stable at their peak locations. The fluctuation range from the nose node to the toe node, after stabilizing, is denoted as $960.03_{-6.53}^{+5.02}$ px, $832.79_{-23.24}^{+12.07}$ px, $760.66_{-11.11}^{+14.27}$ px, $881.06_{-22.06}^{+13.51}$ px, $988.00_{-8.62}^{+5.00}$ px, $1015.16_{-2.94}^{+1.45}$ px, and $1024.51_{-0.56}^{+3.43}$ px. Notably, the shoulder, elbow, and wrist nodes exhibit minor fluctuations after reaching stability.

The vertical velocities of each node shown in Figure 5B reveal that the nose node, neck node, digital node, and toe node stabilize after reaching their peak locations, with fluctuation ranges of $-0.05_{-1.05}^{+1.62}$, $0.40_{-1.28}^{+1.65}$, $-0.04_{-3.18}^{+2.61}$, $0.09_{-6.62}^{+5.49}$, $-0.10_{-2.03}^{+1.85}$, $-0.01_{-1.11}^{+1.06}$, and $-0.04_{-0.97}^{+0.24}$. Similar to the locations in Figure 5A, the velocities of the shoulder node, elbow node, and wrist node fluctuate after reaching their peak positions before stabilizing.

Figure 5C analyzes the joint angles during the cushioning phase, and indicates that the neck joint exhibits the most stable variation, with a range of $173.21_{-16.84}^{+6.52}$. The wrist joint angles, which are associated with the locations of the elbow node, wrist node, and digital node, also demonstrate stable variation. The shoulder joint, elbow joint, and digital joint exhibit the most significant changes, with two distinctive sections. The first section occurs between frames 98 and 156, during which the shoulder joint angles increase rapidly and the elbow joint angles rapidly decrease, while the palm joint angles remain stable. The second section spans frames 157 to 211, where the shoulder joint angles increase slightly, the elbow joint angles start to increase reversely, and the palm joint angles begin to decrease. Remarkably, the section transitions in the shoulder and elbow joint angles correspond to the location and velocity fluctuations in the related shoulder node, elbow node, and wrist node.

The angular velocities of each joint are illustrated in Figure 5D. It is evident that the neck joint holds steady with slight oscillation, ranging from $0.13_{-1.87}^{+1.86}$ °/f. The shoulder and elbow joint angles exhibit significant fluctuations in the first section, with $0.74_{-1.97}^{+2.67}$°/f and $-1.45_{-3.86}^{+3.77}$°/f, respectively. In the second section, the fluctuations decrease to $0.07_{-1.71}^{+1.23}$°/f and $0.30_{-1.16}^{+2.14}$°/f. The wrist joint shows considerable fluctuation, with a range of $-0.13_{-7.04}^{+4.92}$°/f. The fluctuations of the digital joint are smooth at $-0.28_{-1.85}^{+2.39}$°/f, with notable fluctuations only at the beginning of the first section in $1.05_{-2.45}^{+4.17}$ and during the section transitions in $-0.36_{-3.77}^{+2.13}$°/f. 

Based on the video clip and the analysis above, cats achieve smooth landings by controlling their body posture. Throughout the cushioning process, muscular and skeletal control ensures smooth and adaptive joint and muscle responses. This approach reduces the impact of landing, preventing abrupt changes that could harm the internal organs. The shoulder and elbow joints exhibit the greatest variation, indicating their pivotal role in the cushioning process. In contrast, the wrist and digital joints display smaller variations due to their extensive contact with the ground. The shoulder and elbow joints undergo reverse movements during cushioning, which is attributed to the cat’s adjustment of body orientation after fully absorbing the impact of landing. This indicates that the cushioning actions are distinct from the orientation adjustments. The neck joint, along with the associated nodes (nose node, neck node, and shoulder node), experiences the least impact, demonstrating the effectiveness of the cat’s cushioning strategy to reduce and smooth out landing shocks.

### 3.2. Cat Landing Strategy

Furthermore, two additional sets of experiments were conducted (Supplementary Material Section S5). The results show cats exhibit repeatable jumping characteristics during the airborne phase and the impact absorption section of the cushioning phase. Therefore, the cat’s specific cushioning strategies with its forelimbs, from the analysis of kinematic characteristics, are listed as follows.

1. The vertical velocities of each node approach zero once the front legs have completed the entire cushioning process. This indicates that the cat uses its front legs to absorb most of the landing impact during landing.
2. In the airborne phase, the cat fully extends its leg to maximize the capacity of the joint cushioning in preparation for landing. The digital joint and the wrist joint are more active, with their angles continually adjusting to fine-tune the forelimb’s posture. The wrist joint and the digital joint are utilized to modify the paw’s contact angle for landing.
3. In the cushioning phase, the impact absorption and orientational shift are separate actions. The impact is absorbed first, followed by reorientation. The shoulder and elbow joints play a significant role in this phase.
4. Throughout the entire process, there is a logical hierarchy for the joint operation. As the cat needs to fine-tune the posture, the wrist joint and the digital are involved. Meanwhile, the shoulder joint and the elbow joint are primarily used to handle major posture adjustments like impact absorption.
5. The neck joint maintains stability, and varies slightly during the landing process as other joints absorb most of the impact to protect the head.

The cushioning strategies employed by cats can effectively transform the landing strike into a prolonged, low-intensity force, thereby protecting the brain and internal organs within the torso. These strategies enhance the cat’s agility. The forelimbs absorb most of the landing impact, demonstrating the potential for adaptation to robotic platforms. The flexible spine also plays an important role in posture adjustment. Our primary target is to analyze the cushioning strategy in the vertical direction. The subsequent analysis focuses on the vertical direction. 

### 3.3. Simulation of Cushioning Strategy on Robot Platform

#### 3.3.1. Transferring the Strategy onto the Robot Platform

Transferring the cushioning strategy of a cat during its jumping process to a robotic platform is a challenging task. The joint configuration and movements of a cat differ from those of common robotic platforms. Cats have five joints, whereas robots typically have two to balance flexibility and simplicity [48,49]. Simplifications of joint configurations and movements are necessary during the transfer. Meanwhile, it is rare to see cats falling vertically in nature. Most scenarios involve forward-tilted jumps. It is essential to distinguish whether each movement aims to cushion the impact or to rotate the body. 

As mentioned above, the strategy needs to be simplified to better transfer the cat’s cushioning strategy to our robotic platform. The landing process mainly happens on the sagittal plane, while the shoulder yaw joint is used for forward movements, and the wrist yaw joint is employed during hunting or feeding to grasp objects. These movements are in the horizontal and coronal planes, respectively. Therefore, ignoring these two joints, the cat’s five-joint system is successfully reduced into a three-joint configuration. The analyses above indicate that cats prioritize joint movements at different stages. The shoulder and elbow joints primarily handle major posture adjustments, while the wrist and digital joints fine-tune the posture to ensure stable contact with the ground. A feasible approach to simplifying the transfer of a cat’s five-joint system to a robotic platform with three joints is to focus on the cat’s shoulder and elbow joints as primary points of interest, while treating the wrist joint as a follower joint. The robot utilizes the shoulder and elbow joints as the primary control points, mirroring the structure of typical robotic platforms. The wrist joint serves as a passive follower joint connecting to the foot end. This simplified three-joint system effectively replicates the cat’s cushioning strategy. This approach leverages the natural hierarchy and coordination of the cat’s joints, enabling the robot to achieve similar agility and impact absorption capabilities. Moreover, the cat’s cushioning strategy has clear logic at each stage, with well-defined purposes for its movements. During the airborne phase, the primary role of the cat’s joints is to extend its body, gradually transitioning from the take-off posture to the pre-landing posture, and preparing for landing. In the cushioning phase, the cat’s actions to absorb impact and rotate its body during a forward-tilted jump are distinctly separated, which helps to separate movement purposes and retain only the vertical cushioning movements for the robot platform.

**Figure 6:**
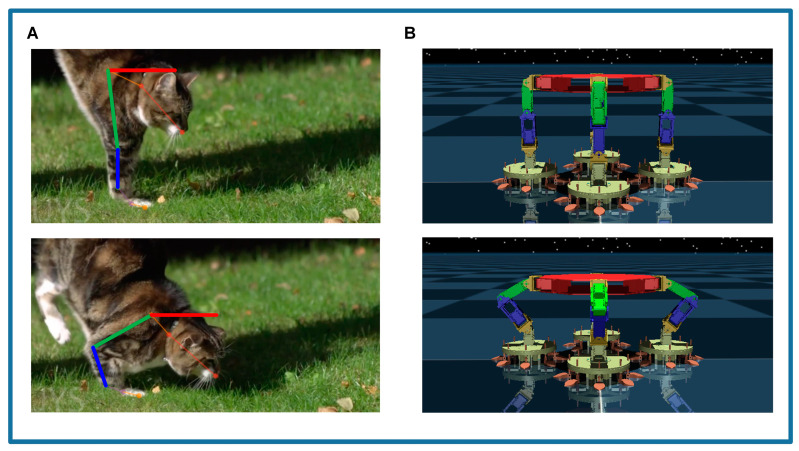
Simulation of the cat’s cushioning strategy on the robot platform. (**A**) The two key frames during the cat’s landing. (**B**) The two corresponding postures of the robot.

Consequently, the cat’s cushioning strategy is simulated on our robotic platform in MuJoCo. The frame in which the cat touches the ground is taken as the robot’s initial posture (frame 97), with joint angles configured at (90.13°, 2.05°, −2.18°). The frame after the cat has completed the cushioning action is taken as the robot’s final posture (frame 156), with joint angles configured as (30.07°, 79.12°, −19.19°), as shown in Figure 6A,B. The quintic polynomial is used to fit the changes in the shoulder and elbow joints during the cushioning process. To ensure that the robot’s velocity reduces to zero after completing the cushioning, the tail-end data is extended using a moving average method. The specific method is detailed in Supplementary Material Section S6. The x-axis represents the time interval for performing the cushioning action. The larger the time interval, the slower the robot executes the cushioning action. The fitting results of the shoulder and elbow joints were obtained. On transfer to the robotic platform, the robot experienced end sliding due to forward-tilted landings (Shown in Viedo S6). To ensure stable contact between the robot’s end effector and the ground throughout the cushioning process, it was necessary to control the wrist joint to ‘align’ the cat’s body. Based on a geometric relationship analysis of the robot’s joints (see Supplementary Material Section S7), the fitted data for the wrist joint were derived. As mentioned above, this approach ensures a smooth and controlled transition from the initial to the final posture, effectively mimicking the cat’s natural cushioning strategy.

#### 3.3.2. Simulating the Cushioning Strategy in Gravity

In the simulation, the baseline is set with an initial height of 0.564 m to achieve an impact velocity of 2.0 m/s. The initial height for the robot landing is set to ensure the robot reaches the desired impact velocity upon contact with the target surface. In actual on-orbit servicing, the carrier spacecraft must approach and fly alongside the target spacecraft before releasing the robot to land on it for servicing. The relative velocity between the robot and the target spacecraft is minimal. The cushioning time interval is 30 times the simulation time step, with each time step being 0.01 ms. Viedos S2 and S3 show the simulation of the robot under gravity, with, and without cushioning strategies, respectively.

**Figure 7:**
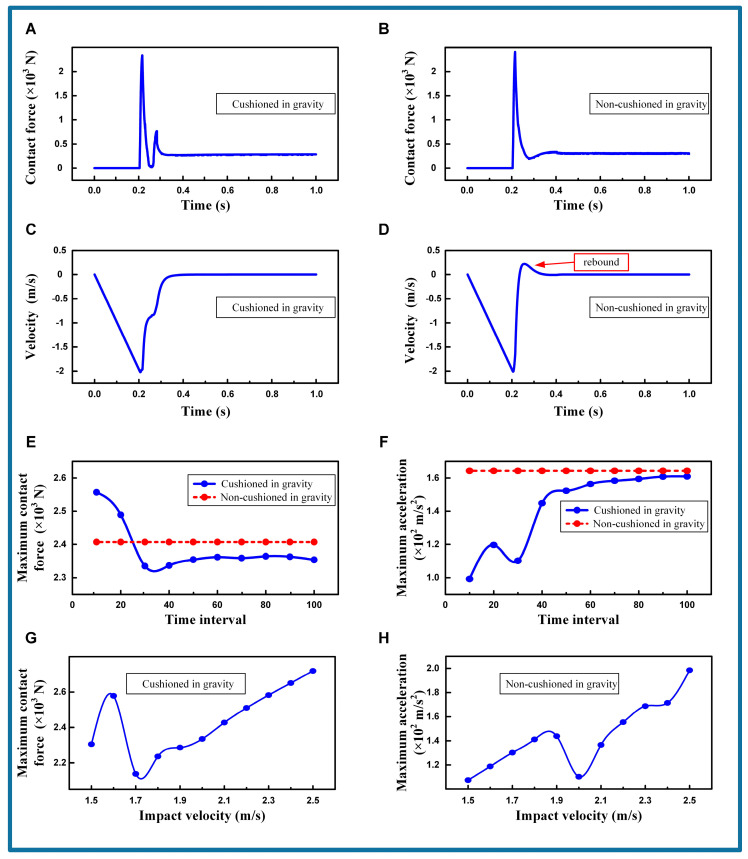
The cushioning characteristic under gravity. (**A**) Contact force is cushioned in gravity. Contact velocity = 2.0 m/s. Time interval = 30. (**B**) Contact force is non-cushioned in gravity. Contact velocity = 2.0 m/s. Time interval = 30. (**C**) Velocity is cushioned in gravity. Contact velocity = 2.0 m/s. Time interval = 30. (**D**) Velocity is non-cushioned in gravity. Contact velocity = 2.0 m/s. Time interval = 30. (**E**) Maximum contact force comparison with time interval. The red dashed line is the non-cushioned situation. (**F**) Maximum acceleration comparison with time interval. The red dashed line is the non-cushioned situation. (**G**) Maximum contact force comparison with impact velocity. (**H**) Maximum acceleration comparison with impact velocity.

Figure 7A,B illustrate the variation in contact forces at the robot’s end with and without the cat-like cushioning action, respectively. Figure 7C,D show the corresponding velocity changes in the robot’s main body under these conditions. It is observed that the cushioning action results in a slight reduction in the maximum contact force at the robot’s foot ends, decreasing from 2407.1 N to 2335.2 N, a reduction of 3%. This indicates that the cushioning action has a negligible effect on the maximum force experienced by the robot’s foot ends. However, the residual stress of cushioning causes the robot to take longer to reach a stable state without a buffer. In contrast, the robot can quickly achieve equilibrium with the buffer, from 220.7 ms to 108.6 ms, a reduction of 50.79%. Considering 5% of the maximum contact force as residual stress, the buffer time surpasses the non-buffer time, from 84.3 ms to 44.7 ms, a reduction of 46.98%. For the robot’s main body, the cushioning action markedly reduces the rate of velocity change, with the maximum acceleration decreasing from 164.4 m/s^2^ to 110.2 m/s^2^, a reduction of 32.97%. Additionally, it eliminates the velocity rebound of the main body, thereby significantly mitigating the impact experienced by the robot’s main body.

The variations in the maximum contact force at the end effectors and the maximum acceleration of the main body under different cushioning time intervals are depicted in Figure 7E,F. The red horizontal lines represent the maximum contact force and acceleration without cushioning. Time intervals refer to the duration between two consecutive control signals in a simulation. They determine the frequency at which the actuator updates its control signals, and influence the speed and accuracy with which the actuator reaches the target position. The curve variations indicate that excessively large or small time intervals cannot accommodate the robot’s landing impact. As the time interval increases, the maximum contact force exhibits a noticeable peak and trough, with the minimum value occurring at 30 times the time step. Beyond 20 times the time step, the maximum contact force experienced by the robot with cushioning is consistently lower than without. The maximum acceleration is obtained by differentiating the velocity during the cushioning process. The velocity is measured with the sensors installed at the end. Moreover, the maximum acceleration shows a fluctuating upward trend as the time interval increases, with local minima at 10 and 30 times the time step. Based on the analysis of maximum contact force, it is concluded that, for an impact velocity of 2.0 m/s, a time interval of 30 times the time step is optimal, minimizing the impact on both the ground and the robot itself.

The variations in the maximum contact force at the foot pads and the maximum acceleration of the main body under different impact velocities (1.5 m/s to 2.5 m/s) are shown in Figure 7G,H. The impact velocity is the speed at which the robot touches the ground. The curve variations indicate the increase in impact velocity and the adaptability of the simulation parameters to the impact velocity. As the impact velocity increases, the maximum contact force at the robot’s foot ends shows a fluctuating upward trend, with local minima at 1.5 m/s and 1.7 m/s. Beyond 1.9 m/s, the maximum contact force increases linearly with the impact velocity. When the impact velocity is between 1.5 and 1.9 m/s, the maximum acceleration of the main body increases linearly with the impact velocity, reaching a peak of 2.0 m/s.

Additional simulations are conducted in Supplementary Material Section S8 to analyze the effects of varying the robot’s mass and surface material on rebound suppression in gravity. The results indicate that the cushioning strategy can effectively reduce the robot’s rebound. In summary, in a gravitational environment, a robot platform employing a cat-like cushioning strategy effectively reduces the impact on its body. Notably, the impact on the main body and the duration of the cushioning time are significantly reduced. In nature, cats use cushioning actions to quickly stabilize their bodies, which is crucial for protecting themselves and enabling agile movements while hunting. The strategy is intuitive and can be applied to optimize the robot’s cushioning effect by adjusting the speed of the cushioning action and the impact velocity.

#### 3.3.3. Simulating the Cushioning Strategy in Zero Gravity

For space applications, simulations are conducted to examine the behavior of a robot in a zero gravity environment, both with and without the implementation of a cat-like cushioning strategy. Initially, the robot is positioned 1 m above the target surface, starting with a velocity of 0 m/s and an acceleration of −9.81 m/s^2^. Once the robot reaches the predetermined velocity, the acceleration is removed, and the robot’s foot ends are equipped with adhesive forces, as detailed in Supplementary Material Section S9. The simulation baseline is set with a target velocity of 2.0 m/s and a cushioning time interval of 30 times the time step, with the other settings mirroring those in the gravitational environment. Viedos S4 and S5 show the simulation of the robot under zero gravity, with, and without cushioning strategies, respectively.

**Figure 8:**
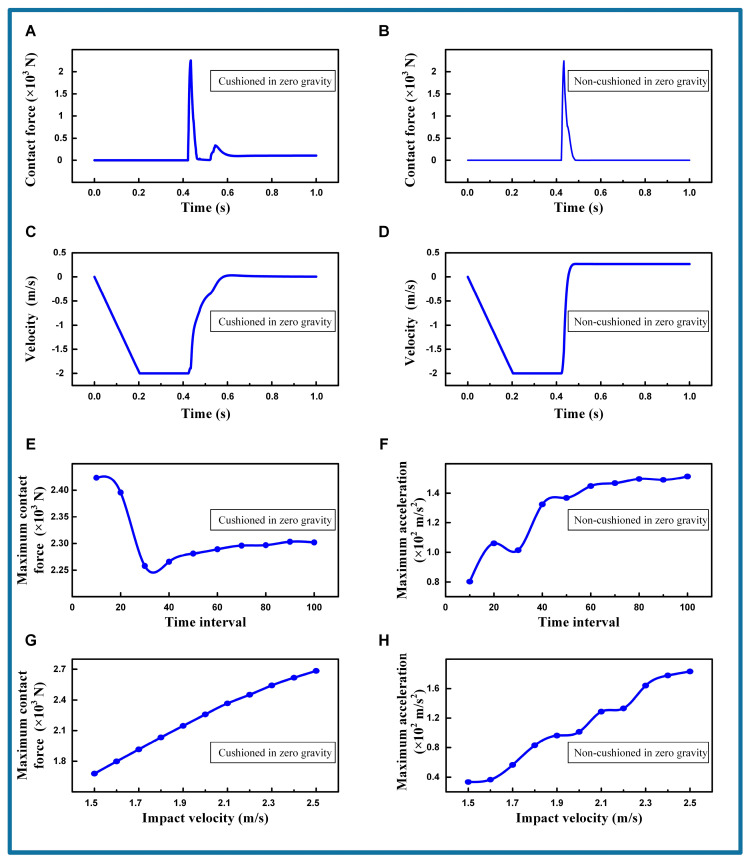
The cushioning characteristic under zero gravity. (**A**) Contact force with cushioned in zero gravity. Contact velocity = 2.0 m/s. Time interval = 30. (**B**) Contact force with non-cushioned in zero gravity. Contact velocity = 2.0 m/s. Time interval = 30. (**C**) Velocity with cushioned in zero gravity. Contact velocity = 2.0 m/s. Time interval = 30. (**D**) Velocity with non-cushioned in zero gravity. Contact velocity = 2.0 m/s. Time interval = 30. (**E**) Maximum contact force comparison with time interval. (**F**) Maximum acceleration comparison with time interval. (**G**) Maximum contact force comparison with impact velocity. (**H**) Maximum acceleration comparison with impact velocity.

In the simulation, the robot without cushioning rebounds upon colliding with the target surface, resulting in a failed attachment, as shown in Viedo S5. The contact forces at the robot’s extremities and the velocity changes in the main body are illustrated in Figure 8B,D. Conversely, when employing the cushioning strategy, the robot stably attaches to the target surface in a zero gravity environment, as illustrated in Viedo S4. The contact forces at the robot’s extremities and the velocity changes in the main body are illustrated in Figure 8A,C. The robot’s foot ends remain attached to the surface without additional vibrations, under the influence of adhesive forces. The robot’s main body retains its velocity after reaching the predetermined speed and gradually decreases to zero upon contact, with no velocity rebound occurring. 

The variations in the maximum contact force at the foot pads and the maximum acceleration of the main body under different cushioning time intervals are depicted in Figure 8E,F. It is apparent that the cushioning time interval still affects the maximum contact force at the foot ends and the maximum acceleration of the main body in a zero gravity state. The maximum contact force at the foot ends initially decreases and then increases with the time interval, reaching a minimum when the interval is 30 times the time step. Simultaneously, the maximum acceleration shows a fluctuating increase as the time interval increases, with local minima also occurring at 10 and 30 times the time step. Based on the analysis of maximum contact force, it is concluded that the impact velocity of 2.0 m/s and the time interval of 30 times the time step are optimal in a zero gravity environment, minimizing the impact on both the target surface and the robot itself. 

Figure 8G,H illustrate the variations in the maximum contact force at the foot ends and the maximum acceleration of the main body across different impact velocities, ranging from 1.5 m/s to 2.5 m/s. As the impact velocity increases, the maximum contact force at the robot’s foot ends rises approximately linearly. The maximum acceleration of the main body also follows an overall upward trend, with local minima observed at 2.0 m/s and 2.2 m/s. Furthermore, the robot consistently adheres to the target surface without rebounding across all tested time intervals and impact velocities.

Additional simulations are conducted in Supplementary Material Section S3 to analyze the effects of varying the robot’s mass and surface material on rebound suppression in zero gravity. The result shows that the cushioning strategy can effectively reduce the robot’s rebound. In summary, employing a cat-like cushioning strategy in a zero gravity environment effectively prevents rebound caused by excessive landing impact. Like the ground environment, the cushioning effect can be further optimized by adjusting the speed of the robot’s cushioning action and the impact velocity, even under zero gravity conditions.

## 4. Discussion

This paper investigates the landing process of a cat’s forelimbs to address the issue of minimizing landing impact for legged space robots during on-orbit operations. The skeletal structure and biomechanical characteristics of the cat are analyzed to identify the necessary forelimb motion chain during landing, which comprises seven nodes and six edges. Using the open-source animal pose estimation tool SLEAP, the motion chain of the cat is tracked throughout the landing process. This tracking provides the positions of various nodes and joint angles at different phases of the cushioning process. Through an analysis of these cushioning actions, the cat’s cushioning strategy is identified. The results indicate that the cat’s cushioning strategy effectively transforms the landing impact into a prolonged period with low-intensity force, thereby protecting the brain and internal organs. By integrating the derived cushioning strategy with the configuration of robotic platforms, reasonable assumptions and simplifications of the cat’s motion chain and cushioning process are formulated. This integration facilitates the successful adaptation and transplantation of the cat’s cushioning strategy to the robotic platform, which is validated using MuJoCo. The results demonstrate that the transplantation of the cushioning strategy needs to consider the robot’s motion parameters. The robot’s cushioning effectiveness is influenced by the speed of the cushioning action and the landing velocity. The optimized cushioning strategy reduces the impact on the ground, extending the cushioning time, and effectively suppresses the robot’s rebound. Furthermore, validation in a zero gravity environment demonstrates that the optimized cushioning strategy is also adaptable to space conditions, effectively reducing the impact on the surface. This highlights the significant adaptability of the cat’s cushioning strategy.

This study focuses on the cat’s landing process to explore the application of its cushioning strategy in the landing control of legged space robots. This research provides a novel bionic control strategy for landing control in space robot on-orbit operations. We believe that the cat’s biological insights into the logical hierarchy for joint operation and the distinct actions between the impact absorption and orientational shift will support the development of future space robots. The Cat’s Landing Control will enhance the in-orbit service capabilities of future robots. This paper showcases the significant expansion of bionics in space environments, further illustrating the promising future of legged robots in space station operations and planetary surface hopping mobility. Our future work will focus on analyzing other animal models with strong jumping abilities, aiming to integrate their bionic features for further impact reduction.

## Data Availability

The data that support the findings of this study are available from the corresponding authors upon reasonable request.

## Supplementary Materials

The following supporting information can be downloaded at: https://www.mdpi.com/article/10.3390/biomimetics9110691/s1, Video S1: the cat’s falling down process; Video S2: the simulation of robot under gravity with cushioning strategy; Video S3: the simulation of robot under gravity without cushioning strategy; Video S4: the simulation of robot under zero gravity with cushioning strategy; Video S5: the simulation of robot under zero gravity without cushioning strategy; Video S6: the sliding situation of the robot without angle adjustments. Reference [50] is cited in the supplementary materials.
