# Supplementary material for: Analysis of Cushioned Landing Strategies of Cats Based on Posture Estimation
Source: Biomimetics (Basel). 2024 Nov 13;9(11):691. doi: 10.3390/biomimetics9110691 (PMC11592395; doi:10.3390/biomimetics9110691)
Supplement: Supplementary file 1 [file biomimetics-09-00691-s001.zip › SuppMater_v2.pdf]

Supplementary Materials

This file includes:

Supplementary Text
Figures S1 to S5
Table S1 to S6

Other Supplementary Materials for this manuscript include the following:

Movies S1 to S6

Supplementary Text

Section S1 The SLEAP parameter settings

The SLEAP parameter settings are shown in Table S1.

Table S1. The SLEAP parameter settings

Parameters	Value	Parameters	Value
Training animal	single animal	Initial Learning Rate	0.0001
Validation fraction	0.1	Max Stride	64
Input Scaling	0.5	Filters	16
Batch Size	24	Filters Rate	2.00
Epochs	1000	Sigma	2.5

Section S2 Calculation of the joint angles

The posture estimation frame SLEAP has been used to extract the position data of each node during the cat's landing process. The joint angle can be calculated with the related three nodes named A, B, and C. The three nodes, A, B, and C, corresponding to joints, are considered to construct vectors in order, and the Angle between the two vectors is calculated by using the dot product formula of the vectors. Take the shoulder joint as an example. The nodes related to the shoulder joint are A (x_1, y_1) for the neck node, B (x_2, y_2) for the shoulder node, and C (x_3, y_3) for the elbow node. The vectors $\overrightarrow{AB}$ and $\overrightarrow{BC}$ can be determined as:

$$\overrightarrow{BA} = (x_1 - x_2, y_1 - y_2)$$
$$\overrightarrow{BC} = (x_3 - x_2, y_3 - y_2)$$

Then, the dot product of the vectors can be got as:

$$\overrightarrow{BA} \cdot \overrightarrow{BC} = (x_1 - x_2, y_1 - y_2) + (y_2 - y_1)(y_3 - y_2)$$

Find the magnitudes of the vectors:

$$\|\overrightarrow{BA}\| = \sqrt{(x_1 - x_2)^2 + (y_1 - y_2)^2}$$
$$\|\overrightarrow{BC}\| = \sqrt{(x_3 - x_2)^2 + (y_3 - y_2)^2}$$

Finally, the angle θis calculated with the arccosine function:

$$\theta = \arccos\left(\frac{\overrightarrow{BA} \cdot \overrightarrow{BC}}{\|\overrightarrow{BA}\| \cdot \|\overrightarrow{BC}\|}\right)$$

$$\theta = arccos\left(\frac{(x_1 - x_2, y_1 - y_2) + (y_2 - y_1)(y_3 - y_2)}{\sqrt{(x_1 - x_2)^2 + (y_1 - y_2)^2} \cdot \sqrt{(x_3 - x_2)^2 + (y_3 - y_2)^2}}\right)$$

The pseudocode for joint angle calculation is as follows:

```
def CalcJointAngle(A, B, C):
    VectorBA = A - B
```

```
VectorBC = C - B
CosAng = dot(VectorBA, VectorBC)/
        (linalg.norm(VectorBA)*linalg.norm(VectorBC))
Ang = degrees(arccos(CosAng))
return round(ang,4)
```

Section S3 Control of the robot's joint with MuJoCo

We created a PD controller to track the joint angle and angular velocity at each joint. The controller consists of a position servo and a speed servo. The position servo has the parameter position feedback gain kp. It can calculate the desired position based on the error between the desired target and the current joint position. The velocity servo has the parameter velocity feedback gain kv. It can calculate the desired velocity based on the error between the desired target and the current joint velocity. We set a relatively large gain parameter to ensure that the robot's joint can quickly track the input angle and angular velocity. kp is set to 500, and kv is set to 200.

As the simulation progresses, the joint angles and angular velocities are input to the corresponding actuator at given time intervals via Python. Time intervals refer to the duration between two consecutive control signals in a simulation.

Section S4 The robotic simulation settings

The robotic simulation settings are shown in Table S2.

Table S2. The robotic simulation settings

Parameters	Value	Parameters	Value
Material	Aluminium alloy	Timestep	0.00001
Density	2720 kg/m^3	Integrator	RK4
Mass	35.25 Kg	Sliding friction	0.8
Joint stiffness	20 N/m	SCRBP stiffness	39 N/m
Joint damping	10 N·s/m	SCRBP damping	16 N·s/m

Section S5 Additional sets of posture estimation

To verify the consistency of the cat's cushioning strategy, two additional sets of posture estimations are conducted using laboratory-raised cats, labeled as Cat 2# and Cat 3#, while the video cat is labeled as Cat 1#. Both cats jumped from a height of 75 cm onto the ground. Figure S1 shows the results of Cats 2# and 3# during the airborne phase. Specifically, Figures S1a and S1b represent the position changes in the X direction, while Figures S1c and S1d represent the position changes in the Y direction. Figure S2 shows the results of Cats 2# and 3# during the cushioning phase. Similarly, Figures S2a and S2b represent the position changes in the X direction, and Figures S2c and S2d represent the position changes in the Y direction. It is evident that there are significant differences in the positions of the three cats in the X direction, indicating that in the horizontal X direction, the cats' cushioning strategies are affected by the actual jumping conditions, resulting in individual differences.

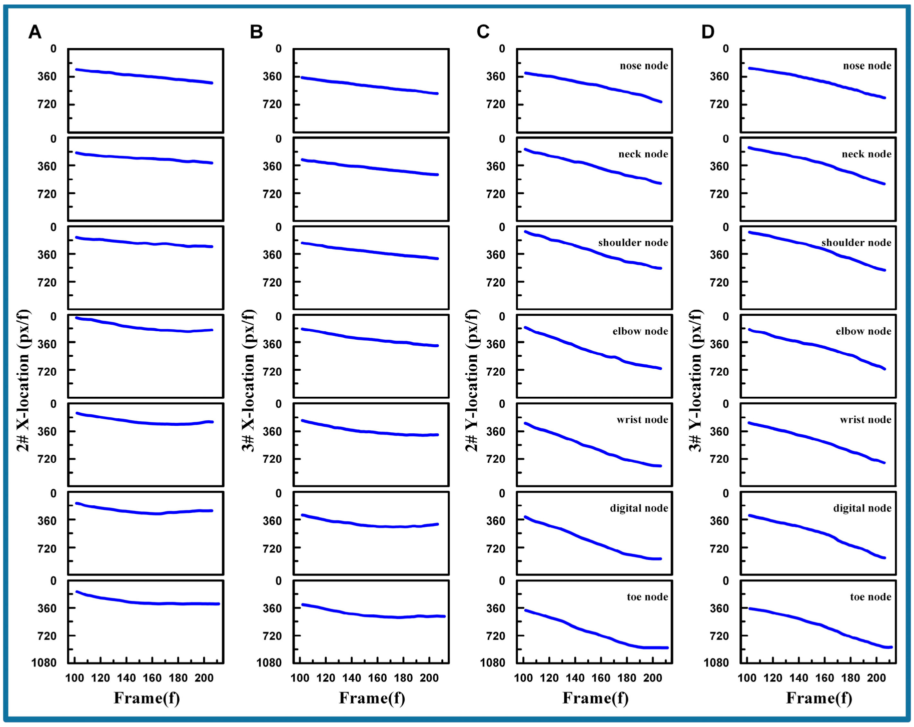

Figure S1. Results of Cats 2# and 3# during the airborne phase. (a) Position changes of Cat 2# in the X direction. (b) Position changes of Cat 3# in the X direction. (c) Position changes of Cat 2# in the Y direction. (d) Position changes of Cat 3# in the Y direction.

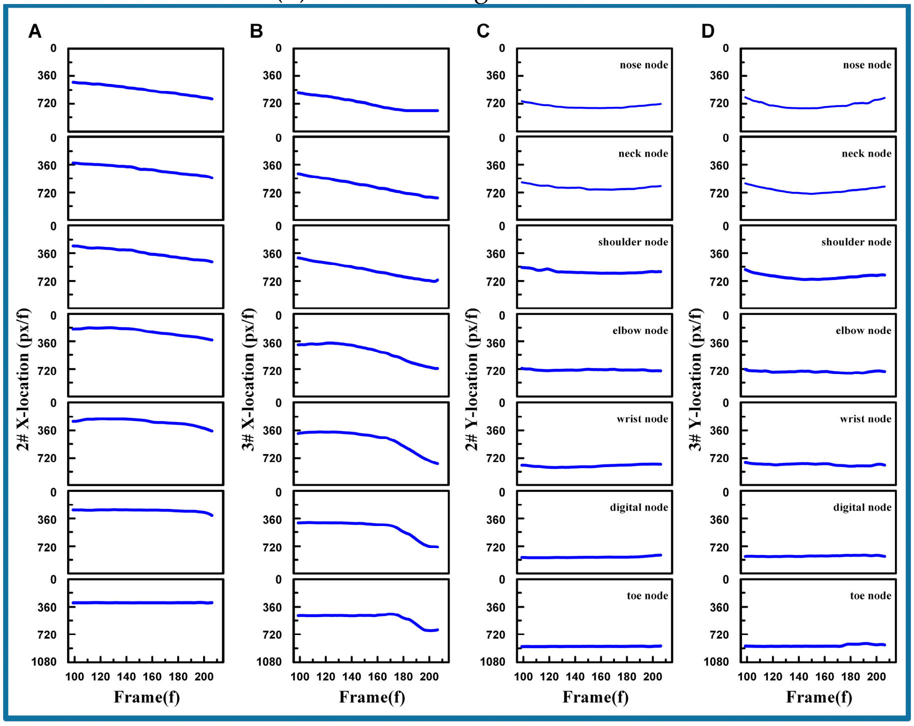

Figure S2. Results of Cats 2# and 3# during the cushioning phase. (a) Position changes of Cat 2# in the X direction. (b) Position changes of Cat 3# in the X direction. (c) Position changes of Cat 2# in the Y direction. (d) Position changes of Cat 3# in the Y direction.

Pearson correlation coefficients are chosen to analyze the similarity in the vertical Y-direction position changes of three cats. Table S1 presents the similarity calculation results for the three cats during the airborne phase, showing that the correlation coefficients of the vertical position curves are all close to 1, indicating a high degree of linear similarity among the three. Table S2 displays the similarity calculation results for the three cats during the cushioning phase. Similarly, the cushioning phase is further divided into two sections, representing impact absorption and orientational shift. In the impact absorption

section, all nodes, except for the palm and digit nodes, maintain a high degree of linear similarity, while the lack of correlation between the palm and digit nodes indicates that these two nodes' movements need to adapt continuously to the target surface during cushioning. In the orientational shift section, there is no similarity among the nodes, indicating that the cats' posture adjustments are influenced by the actual jump, leading to individual differences.

According to the discussion above, it is indicated that the movements of cats during the airborne phase and the impact absorption section of the cushioning phase exhibit similarity in the vertical Y direction, demonstrating the repeatability of these movements. In contrast, in the horizontal X direction and the orientational shift section, the cushioning strategies need to be chosen according to their specific forward postures, resulting in individual differences. The results also validate the analysis of the landing strategy. The wrist and digital joints are involved in fine-tuning the posture, while the shoulder and elbow joints are primarily used to handle major posture adjustments like impact absorption.

Table S3. Similarity calculation results during the cushioning phase.

Nodes	Pearson correlation coefficient		
	**Cats 1# and 2#**	**Cats 1# and 3#**	**Cats 2# and 3#**
Nose	0.997287	0.997546	0.998237
Neck	0.998889	0.996698	0.993715
Shoulder	0.997981	0.99507	0.988886
Elbow	0.997203	0.98261	0.975713
Wrist	0.99808	0.992064	0.987095
Digital	0.997926	0.971629	0.97608
Toe	0.998532	0.982435	0.979157

Table S4. Similarity calculation results during the cushioning phase in impact absorption section.

Nodes	Pearson correlation coefficient		
	**Cats 1# and 2#**	**Cats 1# and 3#**	**Cats 2# and 3#**
Nose	0.987287	0.943704	0.97746
Neck	0.98153	0.985623	0.965218
Shoulder	0.961721	0.966739	0.9601
Elbow	0.843851	0.816087	0.847362
Wrist	0.818936	0.807148	0.778946
Digital	-0.15578	0.156273	0.774251
Toe	0.23715	-0.61526	0.206112

Table S5. Similarity calculation results during the cushioning phase in orientational shift section.

Nodes	Pearson correlation coefficient		
	**Cats 1# and 2#**	**Cats 1# and 3#**	**Cats 2# and 3#**
Nose	0.566104	0.543885	0.985316
Neck	-0.93566	-0.9746	0.967224
Shoulder	-0.82624	-0.77076	0.892371
Elbow	0.765425	-0.19305	-0.68702
Wrist	0.117411	-0.14581	0.042626
Digital	-0.38379	-0.44528	-0.32038
Toe	-0.52221	0.101529	0.284927

Section S6 The fitting method of the joint angles

Based on the data for shoulder and elbow joint variations, as shown in Figure S1a, a fifth-order polynomial is used to fit the variation curves. Since the robot's speed is zero upon landing, it is necessary to ensure that the curvature of the fitted curve approaches zero at the end. Therefore, a moving average method with a window size of 3 was employed to extend the end data of the curve by 20 points.

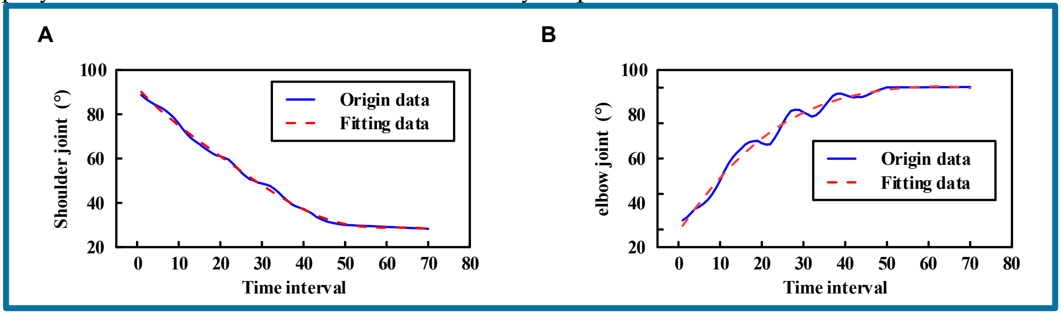

Figure S3. The fitting method of the joint. (a) The fitting results of the shoulder joint. (b) The fitting results of the elbow joint.

The fifth-order polynomial is set as:

$$y = ax^5 + bx^4 + cx^3 + dx^2 + ex + f$$

The fitting results for the shoulder and elbow joints, along with the R^2 values, are presented in Table S1. The R^2 values indicate that the fitted equations can effectively predict the variations in the joint angles.

Table S6. The fitting result of the shoulder joint and the elbow joint.

Parameter	Shoulder joint	Elbow joint
a	-2.25E-07	-8.35E-08
b	3.79E-05	1.41E-05
c	-2.13E-03	-5.96E-04
d	5.74E-02	-3.08E-02
e	-2.12E+00	3.38E+00
f	9.22E+01	-1.30E+00
R^2	0.9978	0.9877

Section S7 The analysis of the robot's geometric relationship

The robot's geometric relationship is depicted in Figure S2. Specify counterclockwise as positive. α, β, and γ represent the shoulder joint, elbow joint, and wrist joints respectively.

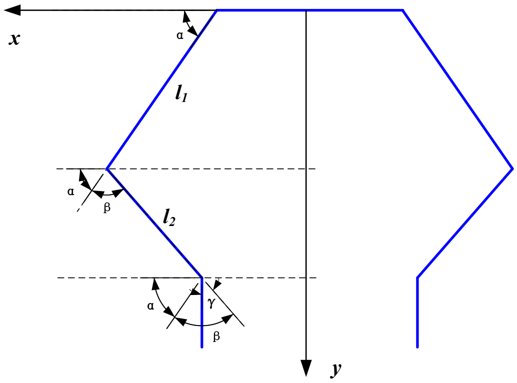

Figure S4. The robot's geometric relationship.

The following relationship of the joints can be obtained as:

$$\alpha + \beta + \gamma = \frac{\pi}{2}$$

Section S8 Analysis of the reduction of the robot's rebound

Figure S3a and Figure S3b show the variation in the velocity under different weights in both gravitational and zero-gravity environments. The robot's weight is set between 25

and 45 kg, with an impact velocity of 2.0 m/s and a time interval of 30 times the timestep. Other parameters remain unchanged. It can be observed that after employing the cushioning strategy, there is no rebound observed in the main body. Figures S3c and S3d illustrate the velocity changes of the robot faced with different surface materials in gravitational and zero-gravity environments. The coefficient of sliding friction for the surfaces is set between 0.5 and 0.9. Other parameters are consistent with the settings above. Similarly, the cushioning strategy prevents rebound in the main body. The result indicates that utilizing the cushioning strategy can effectively achieve rebound suppression and maintain stability upon landing.

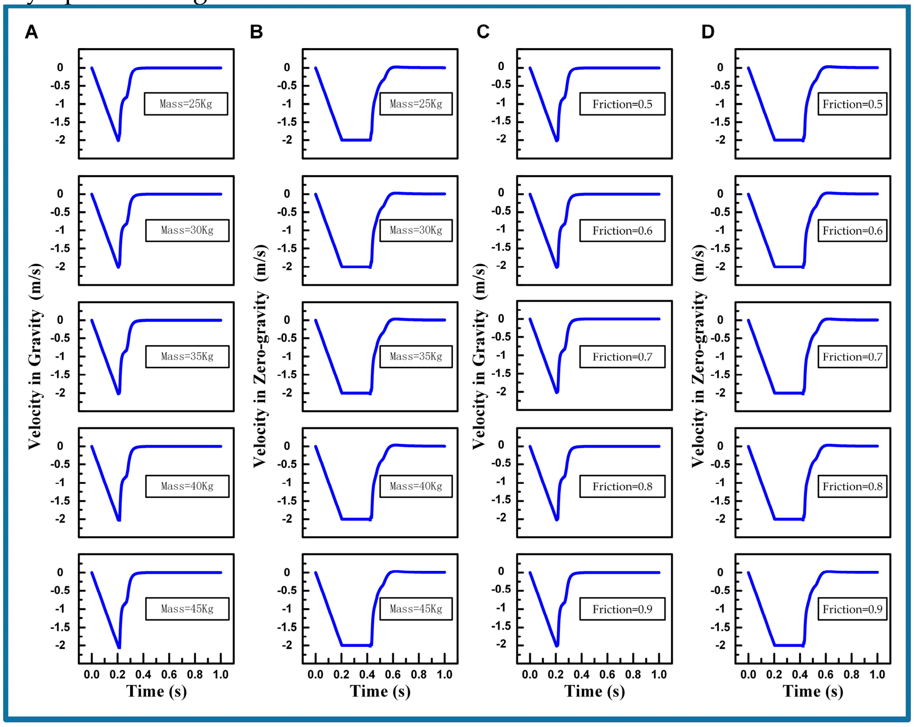

Figure S5. Variation in the velocity. (a) Velocity change of different masses in gravity. (b) Velocity change of different masses in zero-gravity. (c) Velocity change of different sliding frictions in gravity. (d) Velocity change of different sliding frictions in zero-gravity.

Section S9 Calculation of adhesive forces under zero-gravity environment

For space applications, the adhesive force needs to be set at the robot’s foot-end to achieve stable attachment for the target surface. In our previous research, the normal adhesive force at the robot's foot can reach 1.23 N/cm² [1]. Given that the effective area of the robot's foot-end is 169.92 cm², and assuming 50% of the effective contact area is utilized, the adhesive force can be calculated as $169.92 \times 50\% \times 1.23 = 104.5\text{N}$. We defined an adhesion actuator at the robot’s end to inject forces at contacts in the normal direction. The force is divided equally between multiple contacts. Additionally, logical judgment is set in the control Python script. As the robot’s end touches the target surface, the actuator generates the adhesion force.

References

1. Su Y, Hou X, Jiang S, Li M, Liu Y, Yang Zhan, Chen T. Adhesion properties of carbon nanotube arrays for an adhesive foot of a space crawling robot[J]. Smart Materials and Structures, 2019, 29(2): 025001.

Disclaimer/Publisher’s Note: The statements, opinions and data contained in all publications are solely those of the individual author(s) and contributor(s) and not of MDPI and/or the editor(s). MDPI and/or the editor(s) disclaim responsibility for any injury to people or property resulting from any ideas, methods, instructions or products referred to in the content.
